# Image-guided focused ultrasound ablation of breast cancer: current status, challenges, and future directions

**DOI:** 10.1007/s00330-008-0906-0

**Published:** 2008-03-20

**Authors:** A. C. Schmitz, D. Gianfelice, B. L. Daniel, W. P. Th. M. Mali, M. A. A. J. van den Bosch

**Affiliations:** 1 Department of Radiology, University Medical Center Utrecht, Utrecht, The Netherlands; 2 Department of Radiology, University Health Network C/O Toronto General Hospital, Toronto, Canada; 3 Department of Radiology, Stanford University, Stanford, CA USA; 4 Department of Radiology, Lucas MR Imaging Center, 1201 Welch Road, Stanford, CA 94305-5488 USA

**Keywords:** Image-guided therapy, Focussed ultrasound, Breast cancer, Tumour ablation, Magnetic resonance imaging

## Abstract

Image-guided focussed ultrasound (FUS) ablation is a non-invasive procedure that has been used for treatment of benign or malignant breast tumours. Image-guidance during ablation is achieved either by using real-time ultrasound (US) or magnetic resonance imaging (MRI). The past decade phase I studies have proven MRI-guided and US-guided FUS ablation of breast cancer to be technically feasible and safe. We provide an overview of studies assessing the efficacy of FUS for breast tumour ablation as measured by percentages of complete tumour necrosis. Successful ablation ranged from 20% to 100%, depending on FUS system type, imaging technique, ablation protocol, and patient selection. Specific issues related to FUS ablation of breast cancer, such as increased treatment time for larger tumours, size of ablation margins, methods used for margin assessment and residual tumour detection after FUS ablation, and impact of FUS ablation on sentinel node procedure are presented. Finally, potential future applications of FUS for breast cancer treatment such as FUS-induced anti-tumour immune response, FUS-mediated gene transfer, and enhanced drug delivery are discussed. Currently, breast-conserving surgery remains the gold standard for breast cancer treatment.

## Evolution of breast cancer treatment

Breast cancer is the most frequently occurring malignant disease and leading cause of cancer-related death in women in the Western world. The number of new cases and deaths from breast cancer for women in the US in 2007 was estimated to be 178,480 and 40,460, respectively [1]. Radical mastectomy, i.e., breast amputation with or without excision of the pectoral muscle, has long been accepted as an appropriate therapy for breast cancer. This treatment was based on the theory of Dr. William Halsted that aggressive local therapy for control of breast cancer, chest wall and regional lymph nodes would have a substantial benefit on survival [2]. In the 1970s, an increased understanding of the natural history of breast cancer resulted in the use of breast-conserving surgery, i.e., local excision as proposed by Dr. Bernard Fisher, for treatment of breast cancer [3, 4]. After results of randomized studies demonstrated similar survival rates for both treatment groups, breast-conserving surgery combined with radiotherapy became standard treatment for patients with localized breast cancer [3]. Parallel to this development, nationwide breast cancer screening programmes were implemented in many Western countries in the 1990s, resulting in an increased proportion of small carcinomas at time of diagnosis [5]. This further facilitated the implementation of breast-conserving surgery in clinical practice. However, the cosmetic outcome of breast-conserving surgery is often suboptimal, this being due to the fact that the resection of the tumour necessitate a margin of 1 cm normal breast tissue and use of post-operative radiation. Although breast-conserving surgery carries a relatively low morbidity rate, a variety of complications such as bleeding (2%-10%) and infections (1%-20%) can occur [4].

Technological advances over the last decade have fuelled interest in even less invasive treatment of patients with localized breast cancer. Currently available minimally invasive image-guided tumour ablation techniques include radiofrequency ablation, cryoablation, laser ablation, microwave ablation, and focussed ultrasound (FUS) ablation [6]. Different imaging modalities, including fluoroscopy, ultrasound (US), and magnetic resonance imaging (MRI), are used to guide instruments, to monitor the therapeutic procedure and assess treatment response [7, 8]. Compared with traditional surgical methods, minimally invasive image-guided ablation therapies potentially offer several advantages including reduced recovery time and hospital stay, decreased complication risk (infections, bleeding), and the ability to be performed under conscious sedation in an outpatient setting, all factors leading to significant cost reduction [7]. One of the most attractive image-guided ablation therapies is FUS ablation. FUS ablation is a non-invasive procedure, i.e., requires no probe insertion and utilizes focussed ultrasound energy to coagulate tissue [9]. FUS ablation offers a promising method for noninvasive treatment of benign or malignant breast tumours. The breast is an organ with an excellent soft-tissue window that is required for the ultrasound beam to reach the target volume; furthermore, the breast can be easily immobilized. This review outlines the current status and future directions of image-guided FUS ablation for treatment of breast cancer.

## The basic concept of FUS ablation

**Fig. 1:**
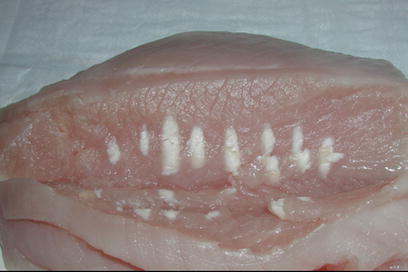
The typical elliptical shape of coagulated tissue after focussed ultrasound ablation

Ultrasound beams are generated by a piezoelectric ultrasound transducer and propagate through tissue as a high-frequency pressure wave. By focussing the ultrasound beams to a focal spot at a certain distance from its source, acoustic energy is converted to heat, and a sharp circumscribed lesion caused by thermal coagulation will be produced [10]. The skin and tissue surrounding the tissue lesion will be unaffected or show negligible temperature rise [11]. The induced tissue lesion has a typical elliptical shape and a volume of 50–300 mm (Fig. 1). For FUS ablation, ultrasound beams with frequencies in the range of 0.5 to 4 MHz are used depending on the application type and penetration depth that is to be attained, i.e., typical penetration depth is 20 cm at 1.5 MHz. The temperature reached within the focal point during a single sonication should be between 60 and 95°C. The mechanism for cell damage is primarily thermal [12]. Rapid increase in tissue temperature above 56°C for 1 s results in immediate protein denaturation and coagulative necrosis. The extent of cellular damage is determined both by the end temperature achieved and length of time for which it is maintained [13]. At high ultrasound intensity levels, in addition to thermal effect, mechanical stresses also occur, resulting in acoustic cavitation and more extensive cell necrosis. In clinical practice acoustic cavitation should be avoided because it may result in unpredictable thermal lesions. Since a single sonication creates a rather small tissue lesion and the need for cooling between sonications necessary to protect tissue heat accumulation and overheating, the time for treatment of a breast tumour of several cm is rather long and ranges from 45 min to 2.5 h. Several recent articles have reviewed the basic physics of FUS for tissue ablation [13–15].

## Choice of image guidance for FUS ablation

**Fig. 2:**
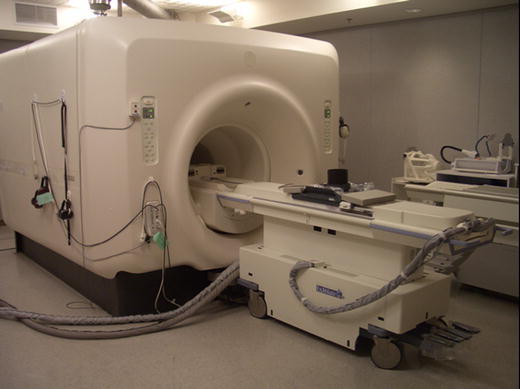
The ExAblate 2000™ FUS ablation device (InSightec, Ltd., Haifa, Israel) with a built-in 3-T MRI system [General Electric (GE) Medical Systems[ in the Lucas MR Imaging Centre, Stanford, CA

**Fig. 3:**
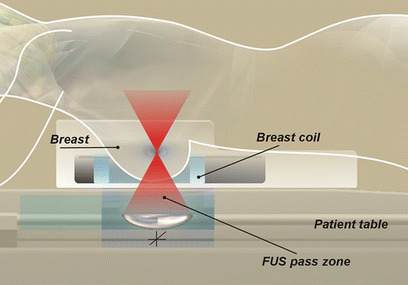
Schematic diagram demonstrating breast cancer patient in prone position and MRI-guided focussed ultrasound equipment

The potential application of FUS ablation for thermal tumour destruction was demonstrated as early as 1942. Lynn et al. reported focal lesions after FUS ablation in mammalian brain tissue [16]. However, initial clinical success of FUS ablation for treatment of neurological conditions and solid tumours was hampered for decades by the lack of reliable treatment monitoring and guidance technology [17–20]. Developments in imaging technology advanced the ability to use FUS effectively, and noninvasive FUS ablation regained widespread scientific momentum in the 1990s after integration of the ultrasound treatment device with modern imaging system. This was achieved in one of two ways, either by using real-time US or MRI [9, 21]. Today, MRI is the most sensitive technique for diagnosing breast cancer with a sensitivity approaching 100%. Furthermore, it is the most accurate imaging modality for visualization and delineation of the breast tumour margins [22–25]. Because MRI has an excellent anatomical resolution and high sensitivity for tumour detection, it offers accurate planning of the tissue to be targeted. An additional advantage of MRI over other imaging techniques is the possibility for MR-based temperature mapping by exploiting the temperature dependence of the water proton resonance frequency [26]. Proton resonance frequency shift thermometry allows monitoring temperature elevations during and after a focussed ultrasound sonication. Damage to adjacent structures can be prevented by evaluation temperature elevations in the surrounding tissue. As a consequence, by combining focussed ultrasound as a noninvasive thermal therapy with MRI for planning, guiding, monitoring, and controlling the focussed ultrasound beam, a real-time image-controlled non-invasive breast tumour ablation system is provided [13, 15]. The commercially available MRI-guided FUS system consists of a FUS ablation device, the ExAblate 2000™ (InSightec, Ltd., Haifa, Israel) integrated in a 1.5-T or 3-T MRI magnet bore [General Electric (GE) Medical Systems, Milwaukee, WI]. The MRI magnet and the focussed ultrasound system are integrated and controlled by a focussed ultrasound workstation and console (Fig. 2). The system provides programmable electronic control over the position and intensity of the focal point across the volume of interest with high flexibility, speed, and precision (Fig. 3).

Diagnostic ultrasound can also be used to guide the FUS ablation procedure [21]. With US guidance, the field of the diagnostic transducer is overlying and arranged parallel to the therapeutic source and offers real-time visualization of the targeted volume [27]. The shortcoming of ultrasound for guiding ablation procedures in the breast is that in general breast tumour size is underestimated with this technique [24]. Accurate delineation of tumour margins is a significant limiting factor of ultrasound FUS guidance as successful ablation of the tumour in its entire volume is mandatory for successful outcome [28, 29]. Another shortcoming of ultrasound guidance during breast tumour ablation is the formation of gas bubbles in ablated tissue. This results in reflection of the ultrasound waves and acoustic posterior shadowing that influences the accuracy for delineation of hypoechoic ablated tissue from hypoechoic breast tumour tissue [28]. Finally, the use of ultrasound during breast tumour ablation is limited by its inability to monitor temperature. Despite these shortcomings ultrasound has been used for noninvasive FUS ablation of breast cancer, although all reports in this area are from the same group [30–38]. Wu and colleagues also developed the only commercially available US-guided FUS ablation system (JC-HIFU system™, Haifu Technology Co. Ltd., Chongqing, China).

## MRI-guided FUS for breast tumour ablation

**Fig. 4:**
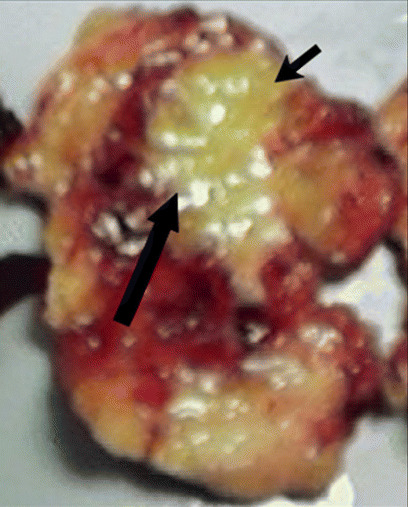
Excised breast tissue specimen after MRI-guided focussed ultrasound ablation of a breast cancer, margins delineated with black arrows

This first experience of MRI-guided FUS for treatment of benign breast tumours was reported by Hynynen et al. in 2001 [39]. They described the use of MRI-guided FUS ablation for treatment of 11 histologically proven breast fibroadenomas. Treatment success was defined as complete or partial lack of contrast material uptake on post-procedural T1-weighted images, which was demonstrated in 8/11 (73%) lesions. In three lesions the treatment was not successful, which was attributed to low acoustic power and patient movement. This report was followed in the same year by the first case report of MRI-guided FUS for treatment of a malignant breast tumour by Huber and colleagues [40]. In this feasibility study a patient with a core biopsy-proven invasive breast cancer was treated with MRI-guided FUS 5 days prior to breast-conserving surgery. Analysis of the breast tissue specimen revealed areas of lethal and sublethal tumour damage, but no exact percentage was provided. After these initial studies, Gianfelice and colleagues were the first to report on the accuracy of MRI-guided FUS for treatment of breast cancer patients, according to a treat-and-resect protocol. In their initial study 12 patients with invasive breast cancer were treated with MRI-guided FUS prior to surgery [41]. Histopathologic analysis of resected tumour in nine patients treated with the newest MRI-guided FUS system showed that a mean of 88.3% cancer tissue was necrosed. Residual tumour was in all cases observed at the periphery of the tumour mass, indicating the need for larger (>5 mm) safety margins around the MRI visible tumour. These results were further supported by findings of the same group in 17 patients with invasive breast cancer that were treated with MRI-guided FUS prior to surgery [42]. Complete (100%) necrosis (Fig. 4) or less than 10% residual tumour was observed in 13/17 (76%) lesions. This study also provided evidence than contrast-enhanced MRI was a reliable method to predict the presence of residual tumour after MRI-guided FUS procedure. In agreement with these findings, Zippel et al. reported the results of a phase 1 trial on MRI-guided FUS ablation of breast cancer, with use of the same ExAblate 2000 system [43]. They treated ten patients 1 week prior to lumpectomy and reported a range of results with complete necrosis in only two patients (20%). Based on this result, it was concluded that there are still several issues that need to be resolved before MRI-guided FUS can become a standard therapeutic technique for breast cancer treatment. They reported that the precision of the focussed sound waves needs to be perfected to destroy 100% of tumour tissue within the lesion, as is achieved with breast-conserving surgery. Secondly, as a surgeon attempts to remove a margin of healthy tissue around the lesion to ensure negative margins, they proposed that it may be necessary to increase the outer limits of the MRI-guided FUS treatment zone to ensure complete eradication of microscopic tumour foci.

In the study performed by Khiat et al. the effect of post-treatment delay for evaluation of MR images on the presence of residual cancer was assessed in 25 patients with 26 tumours [44]. Histopathological analyses following MRI-gFUS showed no residual cancer in 8 lesions (31%), less than 10% in 11 lesions (42%) and between 20–90% in 7 lesions (27%). They recommend an interval of approximately 7 days to determine the effectiveness of MRIgFUS, due to the fact that some benign processes, such as oedema, fibrosis, necrosis, and inflammation, can mimic for a malignant process in that period. More recently, Furusawa et al. published improved results of MRI-guided FUS for tumour ablation in a group of 30 breast cancer patients [45]. All patients underwent surgery after FUS ablation. On pathologic examination the mean percentage of tumour necrosis was 97% of tumour volume. Fifteen patients (50%) had 100% necrosis of the ablated tumour.

The first study on MRI-guided FUS ablation of breast cancer as an adjunct to a preexisting tamoxifen citrate chemotherapeutic regimen for treatment of elderly, high-risk surgical patients was reported by Gianfelice et al. in 2003 [46]. Since no surgical resection was performed after MRI-guided FUS treatment, repeat large core-needle biopsy and contrast-enhanced MRI were used to detect foci of residual tumour. The total follow-up time was 6 months. Overall, 19/24 (79%) patients had negative biopsy results after one or two MRI-guided FUS treatment sessions. All patients completed at least one treatment session. Recently, Furusawa et al. reported on the use of MRI-guided FUS for local treatment of breast cancer in 21 patients: 17 patients were treated once and 4 patients twice [47]. Mean follow-up was 14 months. During follow-up patients underwent 3-monthly breast ultrasound and MRI to detect residual tumour. Based on MRI findings, one case (5%) of recurrence, i.e., mucinous carcinoma, was detected. No evidence of recurrence was detected in the other 20 patients (95%). However, results on the outcome of MRI-guided FUS ablation of breast cancer in non-surgical candidates need to be interpreted with caution, since other adjuvant treatment regimens such as radiation therapy and/or chemotherapy have likely influenced the results. In all studies, MRI-guided FUS treatment was performed under local anesthesia. Patients were given analgesic and sedative agents intravenously to reduce pain, unnecessary motion, and claustrophobia. All patients tolerated the MRI-guided FUS procedure well, and only one minor complication, a skin burn, was reported. An overview of studies on MRI-guided FUS ablation of breast tumours is provided in Table 1.

**Table 1:** Overview of of studies on MRI-guided FUS ablation of breast tumours

	Study	Tumours (n)	Breast tumour characteristics	Outcome of the ablation procedure
1	Hynynen et al. (2001) [39]	11	- Fibroadenomas	- No surgical resection
				- Eight lesions (73%) demonstrated complete or partial lack of contrast uptake (success)
				- Three lesions (27%) showed no marked decrease of contrast uptake (failure)
2	Huber et al. (2001) [40]	1	- Invasive ductal carcinoma (n = 1)	- Surgical resection
				In the treated part of the tumour, cells were partly necrotic and mostly sublethally damaged
				No exact percentage is provided
3	Gianfelice et al. (2003) [42]	17	- Invasive ductal carcinoma (n = 14)	- Surgical resection
			- Adenocarcinoma (n = 2)	- Complete necrosis in four lesions (24%)
			- Infiltrating lobular carcinoma (n = 1)	- Less than 10% residual tumour in nine lesions (52%)
			- All tumours <3.5 cm in size	- Between 30–75% residual tumour in four lesions (24%)
4	Gianfelice et al. (2003) [46]	24	- Breast neoplasms, not specified	- No surgical resection
			- All tumours <2.5 cm in size	- Complete necrosis after 1 or 2 treatments in 19 lesions (79%)
				- Residual tumour after two treatments (failure) in five lesions (21%)
5	Gianfelice et al. (2003) [41]	12	- Invasive ductal carcinoma (n = 11)	- Surgical resection
			- Adenocarcinoma (n = 1)	- Tumour necrosis 43.2% in three lesions (treated with old FUS system)
			- All tumours <3.5 cm in size	- 88.3% tumour necrosis in nine lesions (treated with new FUS system)
6	Zippel et al. (2005) [43]	10	- Breast neoplasms, not specified	- Surgical resection
			- All tumours <3 cm in size	- Complete necrosis in two lesions (20%)
				- Microscopic foci of residual tumour in two lesions (20%)
				- 10% residual tumour in three lesions (30%)
				- Between 10–30% residual tumour in three lesions (30%)
7	Khiat et al. (2006) [44]	26	- Invasive ductal carcinoma (n = 25)	- Surgical resection
			- Infiltrating lobular carcinoma (n = 1)	- Complete necrosis in seven lesions (27%)
			- All tumours <3.5 cm in size	- Less than 10% residual tumour in 11 lesions (42%)
				- Between 20–90% residual tumour in seven lesions (27%)
				- Outcome of one lesion is missing (4%)
8	Furusawa et al. (2006) [45]	30	- Invasive ductal carcinoma (n = 26)	- Surgical resection
			- Ductal carcinoma in situ (n = 3)	- Mean necrosis of targeted breast tumours was 96.9%
			- Invasive mucinous carcinoma (n = 1)	- Complete necrosis in 15 lesions (50%)
			- All tumours <3 cm in size	- Between 95%-100% necrosis in 12 lesions (36%)
				- Less than 95% necrosis in three lesions (4%)
9	Furusawa et al. (2007) [47]	21	- Breast neoplasms, not specified	- No surgical resection
			- All tumours <5 cm in size	- Mean follow-up 14 months (range 3–26 months)
				- Complete necrosis in 20 lesions (95%)
				- One recurrence (5%)

## US-guided FUS for breast tumour ablation

Experience with ultrasound (US)-guided FUS ablation of breast cancer is limited and has only been reported by Wu et al. They reported three studies on the accuracy of US-guided FUS ablation for breast cancer treatment [30, 33, 36]. In the first controlled clinical trial reported in 2003, a total of 48 women were randomized in two treatment groups: (1) the control group in which modified radical mastectomy was performed (n = 25) and (2) the US-guided FUS ablation group, in which modified radical mastectomy was performed within 1–2 weeks following ablation (n = 23) [36]. Of the patients that underwent US-guided FUS ablation prior to surgery short-term follow-up (1–2 weeks), pathologic and immunochemical stains were performed to assess the therapeutic effect of ablation on breast tumour tissue. Pathologic findings revealed that complete necrosis was obtained with US-guided FUS ablation in all patients (100%). The same result on therapeutic effect of US-guided FUS ablation of breast cancer, i.e., 100% necrosis, in the same patients was republished recently [30]. In this most recent report Wu et al. described that a safety margin of 1.5–2 cm around the US visible tumour was chosen to ensure complete tumour ablation [30]. The patient cohort of 23 US-guided FUS-treated patients served as a basis for several other publications mainly focussing on the immunohistochemical results as well [31, 32]. More recently, the same group reported on US-guided FUS as adjunct to chemotherapy, radiotherapy, and tamoxifen for treatment of 22 patients with 23 malignant breast tumours (stage I to IV) [33]. Post-procedural biopsy revealed coagulation necrosis of the entire target tumour in all cases (100%). After a median follow-up of 55 months, one patient died (5%) and two patients (9%) developed local recurrence. The 5-year disease-free survival was 95%. However, since US-guided FUS was used in combination with radiotherapy, chemotherapy, and tamoxifen, it is likely the combination of treatment regimens that resulted in the 5-year survival of 95%. No skin burns or serious complications (bleeding or infection) were observed. It is remarkable that in all US-guided FUS ablation studies a percentage of 100% tumour necrosis was achieved in all patients. In general, this percentage clearly exceeds the success percentage of MRI-guided FUS ablation, although MRI is expected to be the most reliable imaging modality for breast cancer detection and delineation. One potential explanation could be that during the US-guided FUS ablation a larger ablation margin was used (Table 2).

**Table 2:** Overview of of studies on ultrasound-guided FUS ablation of breast tumours

	Study	Tumours (n)	Breast tumour characteristics	Outcome of the ablation procedure
1	Wu et al. (2003)* [36]	23	- Invasive breast cancer, not specified (n = 21)	- Surgical resection
			- Non-invasive breast cancer, not specified (n = 2)	- Complete necrosis in 23 lesions (100%)
			- All tumours <6 cm in size	- Only TTC staining used
2	Wu et al. (2005) [33]	23	- Invasive breast cancer, not specified (n = 21)	- No surgical resection
			- Non-invasive breast cancer, not specified (n = 1)	- Follow-up range 3–60 months
			- Missing (n = 1)	- Complete necrosis initially (2 weeks) in 23 lesions (100%)
			- All tumours <5 cm in size	- Local recurrence (after 18 and 22 months, respectively) in two lesions (9%)
3	Wu et al. (2007)* [30]	23	- Invasive breast cancer, not specified (n = 21)	- Surgical resection
			- Non-invasive breast cancer, not specified (n = 2)	- Complete necrosis in 23 lesions (100%)
			- All tumours <6 cm in size	- Only TTC staining used

*Both studies report on the same patient population treated with US-guided FUS

## Obstacles for clinical implementation of breast FUS ablation

### Breast-conserving surgery is the gold standard

In the past decades, screening programmes and development in breast imaging techniques have led to an increase in the detection of early stage breast cancer [4]. Breast-conserving surgery with radiotherapy has become the gold standard treatment for localized breast cancer [3]. The technique is easy to perform for qualified surgeons and ensures tumour removal. Although breast-conserving surgery is the standard treatment, positive margins are found in between 10% and 53% of the patients [48, 49]. Factors significantly associated with positive margins include large tumour size, younger age, axillary lymph node-positive status, and presence of extensive intraductal component [49]. Furthermore, in breast-conserving surgery, although categorized as a low-morbidity procedure, complications such as bleeding (2%-10%), infection (1%-20%), seroma formation (10%-80%) and chronic incisional pain (20%-30%) occur [5].

To be equivalent to surgical removal, the effect of FUS ablation should be to achieve total (100%) tumour necrosis. Results of studies on FUS breast cancer ablation to date have been variable, with histopathologic analysis demonstrating complete tumour necrosis in 20% to 100% of patients treated [30, 33, 36, 40–47]. Several factors may be responsible for the variable outcome of FUS ablation. Differences in patient selection, imaging techniques used, and tumour ablation protocols are three factors, but one of the most critical factors may be the size of ablated margins, i.e., healthy breast tissue surrounding the tumour that is to be ablated as part of the procedure.

### Size of the ablation margins

Several studies have examined the importance of tumour-free surgical margins after breast-conserving therapy [50]. Ideally the tumour along with a margin of at least 10 mm of normal-appearing tissue is resected to attempt to remove any microscopic cancerous tissue in the region [49]. The minimum cosmetically acceptable tumour-free margin in relation to the risk of local or distant recurrence has been debated in many studies [48, 50–52]. For image-guided FUS ablation successful treatment of the entire tumour relies on accurate tumour volume delineation with the imaging technique (US or MRI) used for targeting and monitoring the ablation procedure. In this respect MRI is most accurate imaging technique currently available [22]. MRI may also underestimate tumour size, especially when an extensive ductal carcinoma in situ (DCIS) component is present [25]. To address the problem of tumour size underestimation and ensure total tumour ablation, different groups have proposed ablation of a margin of “healthy” breast tissue surrounding the breast tumour during FUS treatment. Gianfelice et al. reported in their study that the residual tumour cells were mainly found at the periphery of the treatment field and proposed that this was caused by lack of ablating an adequate amount of surrounding healthy breast tissue around the tumour [41]. Wu et al. treated 1.5–2.0 cm of normal tissue surrounding the tumour and found no residual cancer in all patients [30]. Based on breast-conserving therapy studies, the tumour and a margin of at least 10 mm healthy breast tissue should be ablated to increase the probability of complete tumour necrosis [41].

### Methods for margin assessment and residual tumour detection after FUS ablation

If FUS is to be implemented in clinical breast cancer treatment as an alternative to conventional surgery in the future, a reliable method for tumour margin assessment and detection of residual tumour after FUS ablation must be available. Since FUS treatment would lack a surgically excised pathological specimen, the tumour margin status could not be assessed by histopathological analysis. To date, most protocols have been treat-and-resect protocols, i.e., FUS ablation followed by surgery, which allows histopathological tissue examination to evaluate the therapeutic effect of FUS. In the few FUS ablation studies where treated tumour tissue was left in situ after ablation, different strategies have been used to assess outcome. Furusawa et al. used clinical follow-up by MRI and US every 3 months [47]. If one of the modalities showed suspicious findings suggestive for residual tumour, large core-needle biopsy (LCNB) of the lesion was performed, and if residual tumour or tumour recurrence was found, the affected area was treated again with FUS ablation. Gianfelice et al. used MRI follow-up at 10 days, 1, 3 and 6 months post-treatment to detect residual disease after FUS ablation in high-risk surgical patients [46]. Additional to imaging, after 6 months, multiple LCNBs were performed through different areas of the ablation zone. In case of residual tumour, a second FUS procedure was performed and followed by another targeted LCNB 1 month later. The technique of LCNB for residual tumour detection along the ablation zone margins was also used by Wu et al. They performed LCNB at 2 weeks, 3 months, 6 months and 12 months post-treatment [33]. In case of residual tumour patients were subsequently treated with modified radical mastectomy. Although no definitive guidelines are available today, the combination of contrast-enhanced breast MRI and multiple LCNBs for residual tumour detection appears to be the most promising strategy for candidates after FUS treatment where the treated tumour is left in situ.

### Selection of appropriate patients for FUS ablation

Another important issue for successful FUS ablation is appropriate patient selection. Some breast cancer patients cannot be scheduled for FUS ablation for technical reasons. For FUS treatment a distance of at least 1 cm between the tumour and the skin (to avoid skin burn) and tumour and chest wall (to prevent heat accumulation in the underlying ribs and lung) is required [35]. For MRI-guided FUS ablation, it is also required that, apart from the standard MRI contra-indication, patients must lie still in the closed bore MRI magnet for a long period of time, which can be physically and psychologically difficult, especially in anxious or claustrophobic patients [27]. When FUS ablation is performed for curative treatment of breast cancer, preferentially, patients with large tumours (>5 cm) should not be selected for FUS ablation as increased tumour size significantly increases the treatment time and the probability of complete tumour necrosis decreases substantially [42]. Although there are no guidelines concerning the inclusion of patients based on the tumour size, results from the breast cancer radiofrequency ablation studies showed consistent 100% ablation in patients treated with breast cancers ≤2 cm in size [29, 53]. Another issue that needs to be investigated is the effect of different biological tumour characteristics, i.e., ER/PR and her2neu status of the breast cancer cells, on the outcome of the FUS ablation. So far, no data are available addressing this issue. Additionally, patients diagnosed with extensive DCIS should be excluded from FUS ablation. The diagnostic value of MRI for detecting and predicting tumour size of DCIS is still controversial. Although a recent study showed a high sensitivity of MRI (up to 98%) for the detection of DCIS [54], other study results revealed that MRI tends to under- or overestimate the extent of DCIS tumours in 17–77% and 11–19% of the patients [55–57], hence the use of image-guided FUS ablation may result in overtreatment or more importantly undertreatment of the DCIS component.

When FUS ablation is applied for local tumour control in non-surgical candidates with locally advanced breast cancer or metastatic breast disease, the two issues of tumour size and extensive intradutcal tumour component are less important [46]. In these patients it is important that FUS ablation is a well-tolerated, low-morbidity, and repeatable method for tumour ablation that allows local disease control.

### Impact of FUS ablation on sentinel node procedure

For breast cancer patients, information about the presence of metastases in regional axillary lymph nodes is an important prognostic factor. Sentinel lymph node biopsy (SLNB) is a validated minimally invasive diagnostic procedure used to determine the status of regional lymph nodes for staging purposes [58–61]. Theoretically, FUS might affect the accuracy of the sentinel lymph node procedure by obstructing or alternating the anatomy of breast lymphatics or lymph drainage. Zippel et al. performed sentinel node biopsy in two of their patients treated with FUS [43]. Although a small group, there were no technical problems in finding the sentinel node by the use of contrast agent and radio-isotope-guided surgery. Vargas, et al. conducted a study to determine the success rate of SLNB in patients enrolled in a clinical trial of pre-operative focussed microwave phased-array tumour ablation [60]. Sentinel nodes were found with an overall success rate of 91%, which is comparable with other reports on success rate of SLNB in the literature [58, 59, 61]. Although these results imply that there might be no impairment in the ability to perform sentinel node biopsy after ablation with focussed ultrasound, further investigation is necessary.

## Potential future applications of FUS ablation

Although most of the FUS research focusses on thermal ablation for localized thermal treatment of breast cancer, it has been reported that thermal and mechanical damage of a primary tumour by FUS may trigger systemic biological responses in vivo as well. FUS tumour ablation creates a large amount of tumour antigens in the form of necrotic cells and damaged tumour cells, which may trigger dendritic cell activation. This mechanism may play a critical role in FUS-induced anti-tumour immune response. This hypothesis was recently tested in mice implanted with adenocarcinomas and treated with thermal and mechanical FUS ablation in order to assess FUS-induced effects on the mice’s immunological response, as expressed by in vivo dendritic cell activity [62]. The results confirmed that mechanical FUS ablation resulted in a systemic anti-tumour immune response and that the response is related to dendritic cell activation. The advantage of a systemic anti-tumour immune response is attack of residual tumour cells at the primary treatment site, but also potentially suppression of distant metastases.

Another potential therapeutic application is FUS-mediated gene transfer [63]. One of the key challenges in cancer gene therapy is spatial and temporal control of transgene expression in the tumour cells. In animal models FUS-induced hyperthermia has been successfully used to induce precise focal transgene expression in targeted tumour tissue [64]. This has been achieved by using a heat-sensitive promoter, usually the heat shock protein (hsp)70B promoter and a reporter gene, such as green fluoresecent protein (GFP) or firefly luciferase (Fluc) for in vivo monitoring of accuracy and effectiveness of FUS-mediated transgene expression [65]. Future research will focus on FUS hyperthermia-induced activation of a therapeutic (trans) gene, such as tumour necrosis factor α or interleukin 12.

FUS hyperthermia has also been studied in animal models for targeted chemotherapy to solid tumours. Since conventional chemotherapeutic regimens cause systemic toxicity, developing strategies for improved targeted chemotherapeutic delivery is of great clinical interest. One method used for targeted chemotherapy is the use of heat-sensitive liposomes. In this method the chemotherapeutic drug is encapsulated in the liposome, which results in prolonged intra-vascular circulation time. Because malignant tumours are associated with neo-angiogenesis resulting in leaky vessels, liposomes will preferentially accumulate in the interstitium surrounding the tumour [66]. FUS-induced hyperthermia will enhance both extravasation and drug release when these heat-sensitive liposomes are used [67, 68]. Contrary to these studies that have shown enhanced drug delivery using FUS, results of a recent study assessing FUS for enhanced uptake of liposome-encapsulated doxorubicin in a mouse breast cancer model were disappointing [69]. Further studies are required to investigate the exact mechanisms by which FUS hypethermia may induce targeted drug release and define which therapeutic agents should be used.

## Conclusion

FUS ablation has the potential to become an important modality for non-invasive image-guided treatment of localized breast cancer. Multiple phase I studies have proven MRI-guided and US-guided FUS ablation of breast cancer to be technically feasible and safe. The reported efficacy of FUS ablation as measured by percentages of complete tumour necrosis ranged from 20% to 100%. The difference in outcome between FUS ablation studies can be explained by differences in patient selection, imaging techniques, and tumour ablation protocols used. Although the results of US-guided and MRI-guided FUS ablation are promising, the data are too scant to justify a randomized controlled trial that compares FUS ablation with breast-conserving surgery for treatment of localized breast cancer. To date, MRI-guided treatment protocols of breast tumours offer the most accurate imaging technique for breast tumour targeting, breast tumour delineation, treatment monitoring (including temperature mapping), and detection of residual disease after treatment. The next step towards clinical implementation of FUS for breast tumour ablation would be a large prospective treat-and-resect study that assesses the therapeutic efficacy of MRI-guided FUS ablation in patients with small (<2 cm) solitary breast cancers and provides data that can be used for technique standardization and guideline formulation. In addition, FUS for anti-tumour immune response induction, controlled transgene expression, and targeted drugs delivery in breast cancer patients should be explored. Currently, breast-conserving surgery remains the gold standard for breast cancer treatment.
